# Magnetotail Ion Structuring by Kinetic Ballooning‐Interchange Instability

**DOI:** 10.1029/2021GL096796

**Published:** 2022-02-08

**Authors:** Evgeny V. Panov, San Lu, Philip L. Pritchett

**Affiliations:** ^1^ Space Research Institute Austrian Academy of Sciences Graz Austria; ^2^ Institute of Physics University of Graz Graz Austria; ^3^ CAS Center for Excellence in Comparative Planetology CAS Key Laboratory of Geospace Environment School of Earth and Space Sciences University of Science and Technology of China Hefei China; ^4^ Department of Physics and Astronomy University of California Los Angeles CA USA

**Keywords:** ballooning‐interchange, plasma sheet, ion‐cyclotron waves, finite Larmor radius effect, magnetotail, reconnection

## Abstract

By combining three‐probe THEMIS observations and 3‐D Particle‐in‐Cell simulations, we identify key structures on the ion gyroradius scale that occur in connection with ballooning‐interchange instability heads in the Earth's magnetotail. The mesoscale structures occur at sites of strong ion velocity shear and vorticity where the thermal ion Larmor radius is about half of the width of the head. Finer structures occur at the smaller scales characterizing the wavelength of the electromagnetic ion cyclotron waves generated at the heads. These two processes act to erode and thin the current sheet, thereby forming a local magnetotail configuration that is favorable for reconnection.

## Key Points

- Key ion structures were identified in connection with ballooning‐interchange instability heads in the Earth's magnetotail
- Mesoscale structures were found at strong ion velocity shear/vorticity sites, where the thermal ion gyroradius is half of the head's width
- Finer structures were generated at scales characterizing the wavelength of the electromagnetic ion cyclotron waves

## 1 Introduction

The Solar System has long been extensively used as a natural laboratory to study numerous fundamental processes in collisionless plasmas. One of such processes is irregular gyromotion of ions at any persistent magnetic boundary or transient structure, whose spatial scale is comparable to thermal ion gyroradius. Emerging from such an ion motion, non‐diagonal ion pressure tensor elements drive finite Larmor radius (FLR) effects (Rosenbluth & Simon, 1965; Stasiewicz, 1993), which may result in non‐negligible electric fields due to charge separation.

Being not yet thoroughly investigated theoretically and observationally, these processes are of particular interest at transient northward magnetic field (B_*Z*_) intensifications (dipolarization fronts; DFs) (Nakamura et al., 2002; Russell et al., 1998; Sundberg et al., 2012) in the tails of planetary magnetospheres. DFs are embedded in mesoscale ion bursty bulk flows, or BBFs (Angelopoulos et al., 1994; Baumjohann et al., 1990). Being part of global circulation of the magnetospheric magnetic flux (Dungey, 1961; Vasyliunas, 1983), BBFs/DFs contribute to tailward expansion of the dipolarized region of the magnetotail (Baumjohann et al., 1999; Merkin et al., 2019).

BBFs/DFs are often attributed to magnetic reconnection outflows (Angelopoulos et al., 2008; Coppi et al., 1966) and are indeed found to exhibit prominent FLR effects at the stage of their propagation to and interaction with the dipole magnetic field lines of the Earth's magnetosphere (Pritchett, 2015; Pritchett & Runov, 2017; Sergeev et al., 2009). Alternatively, BBFs could be generated by various interchange processes (Rosenbluth & Longmire, 1957). Confirming this, recent results suggest that kinetic (electron) ballooning‐interchange instability (BICI) (Panov, Nakamura, et al., 2012; Panov, Sergeev, et al., 2012; Panov & Pritchett, 2018a; Pritchett & Coroniti, 2010, 2013; Pritchett et al., 2014) could drive moderate ion flows during detachments of single or multiple BICI heads in the near‐Earth's magnetotail. The latter fact follows from many in situ BICI signatures observed by THEMIS spacecraft that were predicted by Particle‐In‐Cell (PIC) simulations (Panov et al., 2020), as well as from indirect signatures provided by ground All‐Sky‐Imager observations of auroral beads and rays, when single beads or rays lighted up simultaneously with prominent DFs, which were observed by THEMIS spacecraft amidst long chains of azimuthally drifting BICI heads (Panov et al., 2019). Such dipolarization fronts have been interpreted as leading edges of BICI heads detaching from the magnetotail region with reversed B_*Z*_ gradient, the latter being the driver of the BICI instability.

A BICI head detachment progressively builds up ion velocity shears at two head's sides, being part of a dual ion vortex system formed asymmetrically with a larger vortex on the duskside of a dawnward propagating head (Panov et al., 2020). Taking into account typical azimuthal scales of the BICI head and sizes of two side vortices of about 1–2 Earth's radii (R_*E*_) we expect that a significant portion of ions from the magnetotail plasma sheet exhibit agyrotropic rotation in the vicinity of BICI heads. Moreover, such motions should be asymmetric on the dusk‐ and dawnsides of BICI heads due to ***ω* ·** **b** asymmetry of ion FLR effects (Cerri, 2018; Del Sarto et al., 2016; Franci et al., 2016; Huba, 1996; Markovskii et al., 2006; Parashar & Matthaeus, 2016; Yang et al., 2017).

At smaller scales, the BICI heads were found to be rippled by strong off‐equator waves with frequency of the order of the ion gyrofrequency (Panov & Pritchett, 2018b; Pritchett et al., 2014), matching the properties of the left hand polarized and strongly compressional electromagnetic ion cyclotron waves (Perraut et al., 2000). These waves appeared due to strong differential electron‐ion drift propagated azimuthally with speeds of a fraction of *V*_*Ti*_, the ion thermal speed.

Here we investigate ion structures that occur due to agyrotropic rotation of ions and electromagnetic ion cyclotron waves by comparing ions' behavior predicted by the simulation with those collected in situ, when a head developed at the location of three THEMIS probes. We use burst mode three‐point THEMIS (Angelopoulos, 2008) observations on a rare occasion, when the bulk of thermal ion population was well within the operational energy range of THEMIS electrostatic analyzers' (ESA) (McFadden et al., 2008) particle detectors, with ion distribution functions sampled once every 3 s. We further improved the quality of the ion data using measurements from Solid State Telescopes (SST) for energetic ions by combining the ESA and SST distribution functions and ion moments. For our analysis, we used 128 Hz resolution electromagnetic fields data from the THEMIS fluxgate magnetometers (FGM) (Auster et al., 2008) and electric field (EFI) instruments (Bonnell et al., 2008).

## 2 Comparision of THEMIS and PIC Data

**Figure 1:**
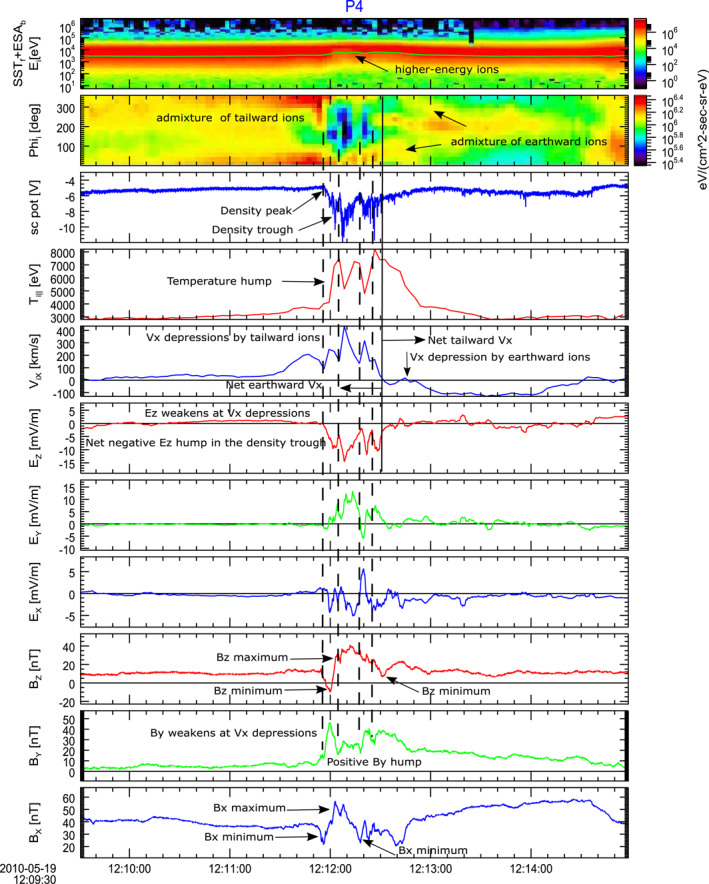
Time History of Events and Macroscale Interactions during Substorms (THEMIS) probe P4 observations on 19 May 2010 between 12:09:30 and 12:15 UT. From top to bottom are shown: joint Solid State Telescope (full mode) and THEMIS electrostatic analyzer (burst mode) ion differential flux spectra, azimuthal (PHI; 0° corresponds to the Earthward direction) angle of ion velocity, spacecraft potential (a high‐resolution proxy to electron density), parallel component of the ion temperature (red), geocentric solar magnetospheric (GSM) V_*X*_ ion velocity component, DSL E_*Z*_ (red), E_*Y*_ (green) and E_*X*_ (blue) electric field components 3s‐long‐sliding‐window averaged, GSM B_*Z*_ (red), B_*Y*_ (green) and B_*X*_ (blue) magnetic field components.

The top panel in Figure 1 shows joint SST (full mode) and ESA (burst mode) ion differential flux spectra as observed by THEMIS probe P4 on 19 May 2010 (Figures S1 and S2 in Supporting Information S1 show the corresponding data for probes P3 and P5 in the same layout). THEMIS data are shown in geocentric solar magnetospheric (GSM) or Despun, Sun‐pointing, L‐momentum vector (DSL) (electric field) coordinates. In the spectra, average ion temperature is overplotted by the green curve. The temperature increased after 12:12 UT, when the lack of lower‐energy ions and admixture of higher‐energy ions was observed (black arrow in the panel). The second panel in Figure 1 shows the ion differential flux distribution depending on the azimuthal (PHI) angle of ion motion. The panel reveals both Earthward (PHI = 0,360) and tailward (PHI = 180) moving ion populations. The dominant Earthward ion population was observed before 12:12:30 UT (denoted by the vertical black line at the time when the ion *V*_*iX*_ velocity component went through zero—cf. fifth panel), which changed to the dominant tailward ion population later. An Earthward‐to‐tailward ion flow reversal is a persistent feature of a BICI head at a later stage of development, which appears due to a dawn‐to‐dusk spacecraft trajectory through the head (Panov et al., 2020).

The Earthward and tailward ion flows were not uniform, as there were also observed minor tailward ion populations before 12:12:30 UT and minor Earthward ion populations after 12:12:30 UT. We show that an admixture of ion populations, which were moving opposite to the direction of the net ion flow, were due to the electromagnetic ion‐cyclotron waves, which may be generated at BICI heads (Pritchett et al., 2014). The minor tailward ion populations were especially pronounced inside a lower‐density interval (density trough) between 12:12 and 12:12:30 UT (see burst mode 128 samples per second spacecraft potential data from EFI in the 3d panel, which is a high‐resolution proxy to the electron density). In the density trough, the ion temperature exhibited an increase of a factor of two with respect to the background ion temperature (temperature hump in the fourth panel). These minor tailward ion populations are associated with the local minima in the temperature hump and with the minima in V_*iX*_ (indicated by four vertical dashed black lines). EFI 3s‐sliding window averaged E_*Z*_ electric field component revealed a negative hump associated with the density trough (negative E_z_ hump in sixth panel; electric field is shown in DSL coordinates, which was close to GSM). This hump is a signature of a current sheet charging. The minor tailward ion populations coincide with local depressions of the negative E_z_ hump (vertical dashed black lines). Two other electric field components (panels 7 and 8) indicate a net duskward E_*Y*_ and variations in E_*X*_. GSM magnetic field components (panels 9–11) reveal larger‐scale variations related to the density trough/temperature hump, and smaller‐scale variations. The smaller scale variations in B_*Z*_ and B_*Y*_ (panels 9 and 10) appeared to be anti‐correlated, and probably related to the minor ion populations (e.g., minima in B_*Y*_ correspond to the minima in V_*iX*_). Similar (but time shifted) larger‐scale variations in B_*Z*_ and B_*X*_ sequentially exhibiting minima, then maxima and again minima (highlighted by the black arrows) coexisted with a broad net positive hump in B_*Y*_ between 12:12 and 12:12:45 UT. B_*X*_ was the main field component (bottom panel), whose variations opposed those in the electron density (third panel). These larger‐scale variations were observed by virtual spacecraft in the following BICI simulations.

**Figure 2:**
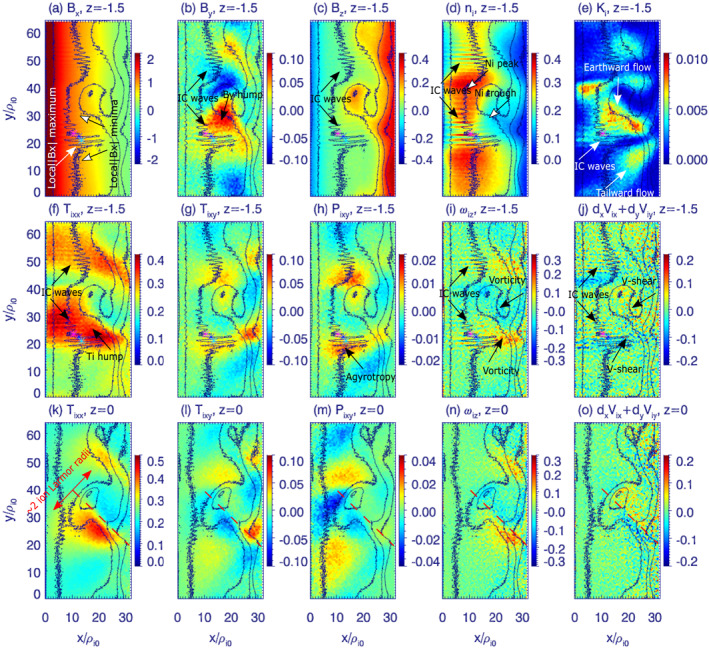
The later‐stage (Ω_*i*0_ *t* = 210) development of the ballooning‐interchange instability in the xy (equatorial) southern hemisphere (*z* = −1.5) and neutral (*z* = 0) planes with the emergence of two ballooning‐interchange instability heads predicted by 3‐D Particle‐In‐Cell simulation using the electron (charged) current sheet. The x coordinate is directed tailward. The y coordinate is directed toward dawn. The coordinates are expressed in *ρ*_*i*0_—the ion gyroradius in the asymptotic lobe *B*_0_ field. The black contours show levels of B_*z*_ in each panel. The following field and plasma quantities are shown in the figure: three magnetic field components (a, b, c), ion density (d), ion kinetic energy (e), ion temperature tensor T_*xx*_ and T_*xy*_ elements (f, g), P_*xy*_ ion pressure tensor element (h), out‐of‐plane component of curl of the ion velocity (i), equatorial component of divergence of ion velocity (j). Panels (k) through (o) show the same simulation quantities as panels (f) through (j) at *z* = 0. The dashed red lines in the bottom row approximately divide the ballooning‐interchange instability head into dusk (below the lines) and dawn (above the lines) parts.

We use the PIC simulation run of a BICI head, for which the setup conditions were explained in Appendix 1 of Panov and Pritchett (2018b); reproduced in PIC Simulation Setup Section in the Supporting Information S1. Figure 2 shows instantaneous development of various field and ion quantities at Ω_*i*0_ *t* = 210, where Ω_0*i*_ is the proton gyrofrequency. The most pronounced BICI head is seen around y/*ρ*_*i*0_ = 30 as a reddish B_*z*_ spot (panel c), being partly detached from the tailward B_*z*_ gradient (20 < *x*/*ρ*_*i*_ < 27). This head drifted dawnward and grew Earthward (cf. Movie S1 for a continuous development between Ω_*i*0_ *t* = 161 and Ω_*i*0_ *t* = 280). Duskward from the head (around y/*ρ*_*i*0_ = 20) were observed electromagnetic ion‐cyclotron waves, driven by the ion‐electron differential drift (Panov & Pritchett, 2018b; Pritchett et al., 2014); the drift is not shown in the present paper. Similar waves (around y/*ρ*_*i*0_ = 50) belong to the duskside of the less pronounced head around y/*ρ*_*i*0_ = 60. There were also observed B_*x*_ and B_*y*_ variations associated with both, the BICI head and the ion‐cyclotron waves, as seen in panels (a, b). A density enhancement and depression (panel d) was observed in front and behind the reddish B_*z*_ spot (N_*i*_ peak and trough). At this stage, the ion‐cyclotron waves significantly modify the structure of the ion flow on the duskside of the head between the Earthward and tailward flows (panel e). In this place, there was also an increase in the ion temperature (*T*_*i*_ hump in panel f). Associated with the hump, non‐diagonal ion temperature tensor T_*xy*_ (g) and ion pressure tensor P_*xy*_ (h) elements being the result of anisotropy in ion velocity distribution function revealed significant agyrotropy in the ion motion caused by decoupling of ion motion from the magnetic field. The agyrotropic ion motion occurred at the locations of ion flow vorticity (panel i), and of the ion flow divergence (panel j). The bottom row in Figure 2 repeats its middle row for *z* = 0 (instead of *z* = −1.5). Although the results are similar to those in the middle row, the quantities at *z* = 0 exhibited more axial symmetry on the two sides of the head: above and below the dashed red lines. The explanation for this is that the ion patterns at *z* = −1.5 were more duskward than those at *z* = 0, which is also seen in Figure 4d below. T_*xy*_ and P_*xy*_ appeared to be the dominant non‐diagonal temperature and pressure tensor elements at *z* = 0 (not shown). According to Figure S6b in the PIC Simulation Setup Section of the Supporting Information S1, the equatorial *B*_*z*_ magnetic field was ∼0.1 of the asymptotic lobe *B*_0_ field (for *x*/*ρ*_*i*0_ between 10 and 20), suggesting the ion gyroradius in the equatorial plane ∼10*ρ*_*i*0_. Hence, the width of the simulated BICI head was about 2 ion Larmor radii in the equatorial *B*_*z*_ magnetic field (Figure 2k).

**Figure 3:**
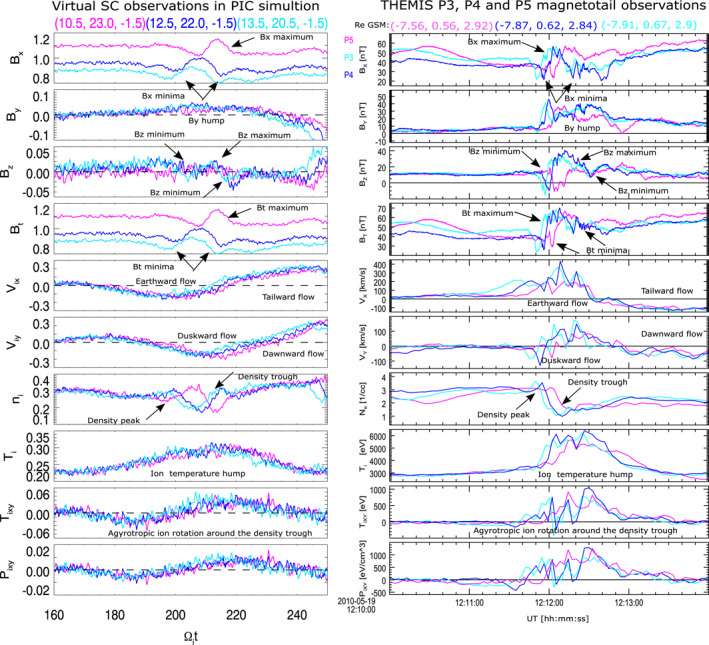
Comparison of ballooning‐interchange instability (BICI) head observations by three virtual spacecraft in the 3D Particle‐In‐Cell simulation box (left column) and by three Time History of Events and Macroscale Interactions during Substorms (THEMIS) probes in the Earth's magnetotail (right column). Locations of the virtual spacecraft and THEMIS probes are provided in tops of the two columns. Results from 3D PIC simulation of BICI development in the electron (charged) current sheet between Ω_*i*0_ *t* = 160 and Ω_*i*0_ *t* = 250 are (left column; from top to bottom) three magnetic field components and the total magnetic field (B_*x*_, B_*y*_, B_*z*_, B_*t*_), X and Y ion velocity components V_*ix*_ and V_*iy*_, ion density N_*i*_, average ion temperature T_*i*_, non‐diagonal ion temperature and ion pressure tensor elements T_*ixy*_ and P_*ixy*_. In the simulation, the *X* axis is directed antisuward and the *Y* axis is dawnward, which is opposite to the GSM *X* and *Y* axes. The three curves correspond to the location of three virtual spacecraft at (x/*ρ*_*i*0_,y/*ρ*_*i*0_,z/*ρ*_*i*0_) = (10.5,23.0,−1.5) (magenta), at (x/*ρ*_*i*0_,y/*ρ*_*i*0_,z/*ρ*_*i*0_) = (12.5,22.0,−1.5) (blue) and at (x/*ρ*_*i*0_,y/*ρ*_*i*0_,z/*ρ*_*i*0_) = (13.5,20.5,−1.5) (cyan). Figure S3 in Supporting Information S1 shows the simulation data at the same X and Y positions, but in the opposite Z‐location (*z* = 1.5). Three THEMIS probes' observations on 19 May 2010 between 12:10 and 12:14 UT by probes P3 (cyan), P4 (blue) and P5 (magenta) (right column; from top to bottom) are three geocentric solar magnetospheric (GSM) and total magnetic field components (B_*X*_, B_*Y*_, B_*Z*_, B_*T*_), X and Y (GSM) ion velocity components V_*X*_ and V_*Y*_, electron density N_*e*_ (we assume quasi‐neutrality of plasma here; the ion density from the joint THEMIS electrostatic analyzer‐Solid State Telescope data underestimated the actual plasma density by up to 20%), average ion temperature T_*i*_, non‐diagonal ion temperature and ion pressure tensor elements T_*XY*_ and P_*XY*_.

With the help of Figure 3 we compare the three‐point THEMIS observations (right column) from Figure 1 and Figures S1 and S2 in Supporting Information S1 with virtual spacecraft observations at three locations in the PIC simulation domain (left column), that are indicated by the cyan, blue and magenta glyphs in each panel of Figure 2. The exact locations of the three glyphs are indicated on the top of the left column. Due to the opposite direction of the *x* and *y* axes in the simulation box as compared to the GSM coordinates, the three virtual spacecraft observations are shown for the southern hemisphere. The corresponding virtual spacecraft observations in the northern hemisphere are provided in Figure S3 in Supporting Information S1. The right column shows data from three THEMIS probes on 19 May 2010 between 12:10 and 12:14 UT. In each panel cyan curve stands for P3, blue—for P4 and magenta—for P5. Probes' locations in R_*E*_ GSM are provided on the top of the column. The barycenter of the probes was near (−8; 1; 3) R_*E*_ GSM. Radially, P5 was about 2,000 km more Earthward than P3/P4; azimuthally P4 was between P3 on the duskside and P5 on the dawnside. Such configuration of the three probes is close (in relative probe location, inter‐probe scales and timing/sequence of magnetotail observation) to those of the virtual spacecraft indicated by three glyphs in Figure 2.

The most pronounced structure (seen in the B_*X*_ GSM magnetic field component; first panel in the right column in Figure 3) reveals a local B_*X*_ peak around 12:12:15 UT, which was surrounded by two minima before and after the peak. This major structure was clearly reproduced in the simulation B_*x*_ on the left, as was the net positive hump in the simulation B_*y*_ as in the observed B_*Y*_ GSM on the right (second panels). The smaller scale shape of B_*Y*_ and B_*Z*_ GSM (second and 3d panels on the right) curves appeared to be anticorrelated (maxima in B_*Y*_ correspond to minima in B_*Z*_ and vice versa). Due to numerical noise, these smaller scale variations were difficult to reproduce in simulation B_*y*_ and B_*z*_. The larger scale behavior of the simulation B_*z*_ still reproduced the maximum and minima features in B_*Z*_ GSM on the right. The total magnetic field *B*_total_ (*B*_*t*_ and *B*_*T*_ in the fourth panels from the top) repeated the main features of B_*X*_ (a maximum between two minima). From the magnetic field, we were able to identify the propagation velocity of the B_*T*_ maximum in the THEMIS data with the help of the timing method (Russell et al., 1983), which was applied to the sharp magnetic boundary in B_*T*_ data around 12:12 UT using time delays in observation of this feature between three THEMIS probes. The magnetotail B_*T*_ maximum appeared to move mainly dawnward along the unit vector *N*_*XY*_ = (0.014; −0.999; GSM), at a velocity *V*_*N*_ ≈ 70.9 km/s. Knowing V_*N*_ one can evaluate the azimuthal scale of the head of about 2,130 km (the duration of the B_*T*_ maximum was ∼30 s). The ion V_*X*_ and V_*Y*_ velocity components (fifth and sixth panels in Figure 3) exhibited Earthward and duskward flow around 12:12:15 UT. Around 12:12:45 UT V_*X*_ and V_*Y*_ reversed signs.

The dawnside of the BICI head in the simulation (which was observed before Ω_*i*_ *t* ≈ 215) appeared to contain a denser and cooler plasma (panels 7 and 8 in the left column of Figure 3), whereas the duskside exhibited lack of plasma and enhanced ion temperature (after Ω_*i*_ *t* ≈ 215 in the same panels). Due to delay of more than 30Ω_*i*_ *t* in virtual probes observation of the head's dawn‐ and duskside, the temperature on the duskside changed more dramatically from the initial ion temperature than on the dawnside. A similar density trough with an ion temperature hump was sensed by THEMIS around 12:12:30 UT (panels 7 and 8 in the right column of Figure 3). The last four panels at the bottom of Figure 3 show xy temperature and pressure tensor elements. Both virtual spacecraft and THEMIS probe observations consistently exhibited significant non‐diagonal ion temperature and ion pressure tensor elements around the temperature hump (simulated T_*xy*_ and P_*xy*_ features appeared periodically together with arrivals of new heads). All THEMIS signatures indicated in the right column were accurately reproduced by the PIC simulations in the left column (disturbances related to the ion‐cyclotron waves were not indicated in Figure 3, because they were not well seen in the simulation data due to numerical noise).

**Figure 4:**
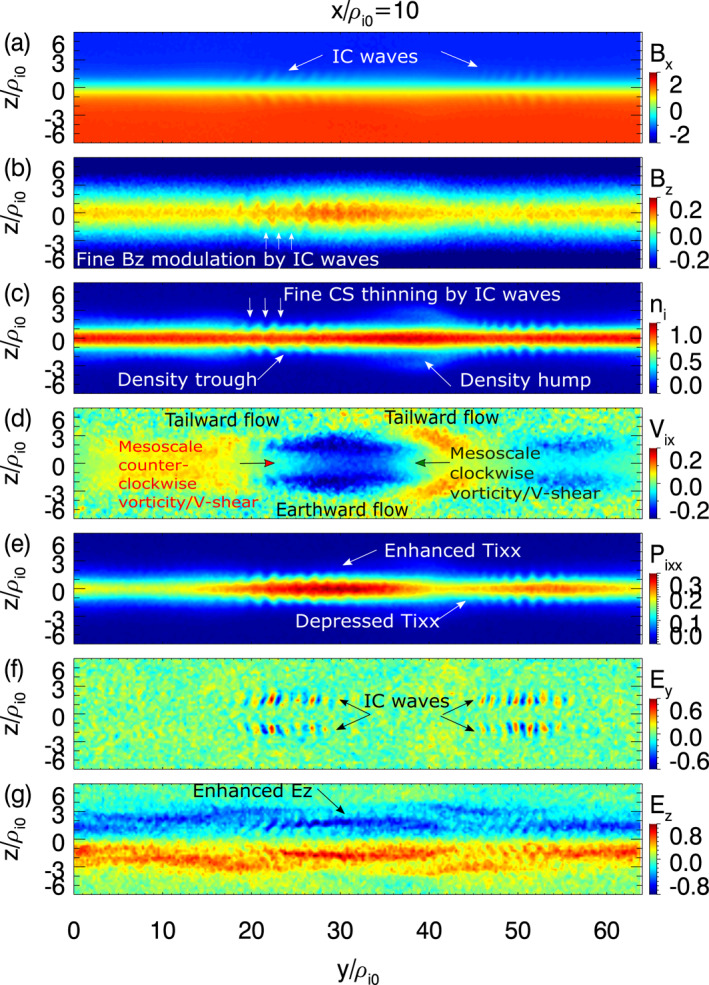
Results from the same 3D Particle‐In‐Cell simulation as in Figure 2 in the yz plane at x/*ρ*_*i*0_ = 10: B_*x*_ (a) and B_*z*_ (b) magnetic field components, ion density (c), ion velocity V_*ix*_ component (d), P_*xx*_ ion pressure tensor component (e), E_*y*_ (f) and E_*z*_ (g) electric field component. The coordinates are expressed in *ρ*_*i*0_—the ion gyroradius in the asymptotic lobe *B*_0_ field.

Figure 4 shows simulation results in the yz (cross‐tail) slices at x/*ρ*_*i*0_ = 10. The middle head is seen in the pronounced Earthward flow (bluish V_*ix*_ spot in panel d), which was drifting to the right (dawnward). The places of mesoscale velocity shears are present on the two sides of the Earthward flow between the bluish and yellowish spots. The density trough (panel c) containing hotter ion population (and larger pressure, panel e) appears at the duskside of the Earthward flow. It is this region, which was observed as peaks in B_*x*_ and B_*t*_ in Figure 3, which gets thinner (also with time) than the surrounding current sheet. A further thinning of this region at smaller scales was provided by the ion‐cyclotron waves, which were localized between the neutral sheet and the lobes around z/*ρ*_*i*0_ = ±1.4. In these locations, the waves modulated B_*z*_ (b), ion density and pressure (c, e), as well as E_*z*_ (g). The appearance of both the density trough and the ion‐cyclotron waves acted to create regions, in which the current sheet tended to become locally one‐dimensional (with a decreased B_*z*_). Complementary to panels (f, g) in Figure 4, Figure S4 from the Supporting Information S1 shows the xy slices of the E_*y*_ and E_*z*_ in the same format as panels in Figure 2. Figure S4 in Supporting Information S1 reveals the radial extent of the electric component of the ion‐cyclotron waves (E_*y*_) and the distribution of the current sheet charging (E_*z*_). The ion‐cyclotron waves also produce ion velocity shears on the scales of the wave length (horizontal stripes in Figure 2e produced by both V_*ix*_ and V_*iy*_).

**Figure 5:**
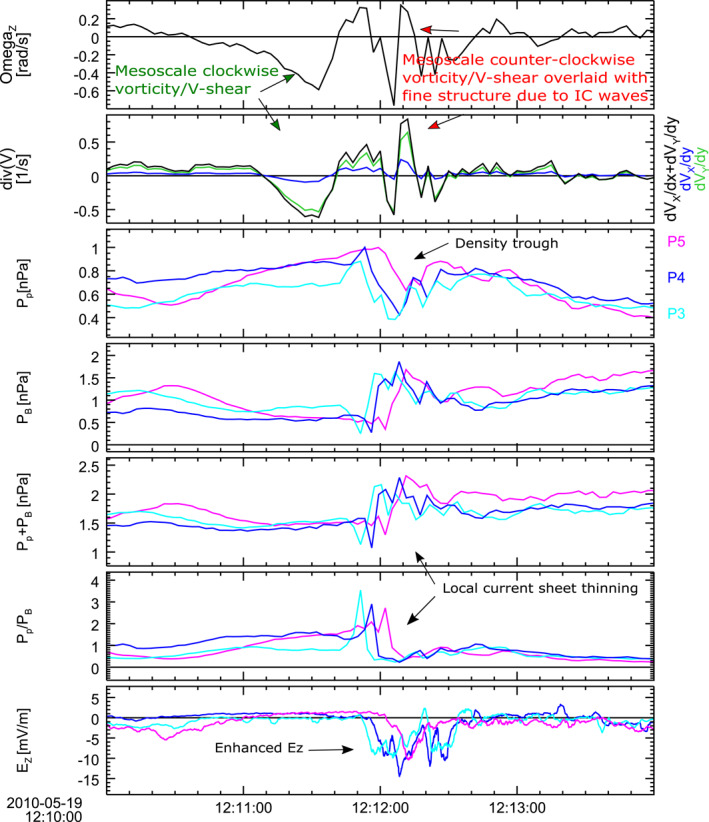
Time History of Events and Macroscale Interactions during Substorms (THEMIS) probes P3–P5 observations on 19 May 2010 between 12:10 and 12:14 UT. From top to bottom are shown: z component of curl of the ion velocity *ω*_*z*_ and equatorial components of divergence of the ion velocity – *∂V*_*ix*_/*∂x* (blue), *∂V*_*iy*_/*∂y* (green) and the sum of the two (black). *ω*_*z*_, *∂V*_*ix*_/*∂x* and *∂V*_*iy*_/*∂y* were calculated for the area of the triangle with its apices at the locations of P3, P4, and P5 (coordinates are provided in the top of the right panel in Figure 3; the triangle is shown in Figure S3 of the Supporting Information S1). Ion pressure P_*p*_, magnetic field pressure P_*B*_, sum of the ion and magnetic field pressures, ion‐to‐magnetic pressure ratio (plasma *β* = P_*p*_/P_*B*_), vertical component of the electric field E_*Z*_ at probes P3 (cyan), P4 (blue) and P5 (magenta). All shown parameters are derived from joint THEMIS electrostatic analyzer‐Solid State Telescope ion observations.

Figure 5 is aimed at identification of regions with stronger vorticity at two heads' sides that are indicated in panel (d) of Figure 4. The top and the second panels in Figure 5 show the Z GSM component of the ion vorticity *ω*_*Z*_ and components of the ion divergence (of which *∂V*_*iy*_/*∂y* appeared to be the major component; green curve), which were calculated using three spacecraft P3–P5 data. A clear and prominent region of the mesoscale clockwise vorticity and velocity shear was observed at the dawnward side of the head (before 12:11:40 UT). As the head drifted dawnward, the three probes started to observe the duskward side of the head. In addition to an average counterclockwise sense of rotation of the ion flows after 12:11:40 UT, the probes also observed significant flow variability of a smaller duration. These smaller scale variations in *ω*_*Z*_ and div(*V*) are related to the minor tailward‐moving ion populations shown in the second panel of Figure 1. They can be explained by the presence of the ion‐cyclotron waves on the duskside of the head. Starting from the third panel, the color coding of Figure 5 is the same as in the right column of Figure 3. This part of Figure 5 allows to understand the observed current sheet dynamics in the location of the density trough (third panel in Figure 5). In the trough, both the magnetic field pressure (fourth panel) and the sum of the magnetic and the plasma pressure P_*p*_ + P_*B*_ (fifth panel) significantly increased, leading to a drop in the plasma‐to‐magnetic pressure ratio (plasma *β* = P_*p*_/P_*B*_; sixth panel). Thus, the trough appeared to be formed inside a locally thinning current sheet, with charging being accumulated (cf. E_*z*_ in seventh panel in Figure 5). Taking P_*p*_ + *P*_*B*_ = 1.6 nPa and *B*_*T*_ = 50 nT at 12:10UT, the magnetotail lobe field *B*_0_ was ∼63 nT, and the ion gyroradius in the lobe magnetic field ∼88 km (assuming *T*_*i*_ = 3 keV), being an observational analogue of *ρ*_*i*0_ in the simulation plots. A lower estimate of the equatorial ion gyroradius of ∼554 km comes from the observed B_*Z*_ ∼10 nT.

After the present THEMIS observations (cf. Figure S5 in Supporting Information S1) we identified two additional heads (denoted as BI head 2, 3 around 12:15:40 UT and 12:17 UT), in addition to the present head (denoted as BI head 1 around 12:12 UT). Both additionally identified heads exhibit less pronounced, but still easily recognizable signatures of the present head, such as the density troughs, the temperature humps, the Earthward flows and the magnetic field variations, being consistent with the multiple appearance of the BICI heads at different stage of development in the simulation (cf. Figure 2, around y/*ρ*_*i*0_ = 58; we could not identify the ion‐cyclotron signatures in the ion data for the additional BI heads, probably due to insufficient time resolution of the data).

## 3 Conclusive Discussion

Ions operate in BICI processes at two scales. Mesoscale ion dynamics was observed as agyrotropic ions' rotation at the head of a cross‐tail size of the order of the thermal ion gyroradius. This ion behavior is presumably stipulated by vorticity and flow shears at the sides of the head. Both observations and the simulation indicate humps (reductions) in the ion temperature (density) on the duskside of the heads, and ion temperature reductions (density humps) on the dawnside. The humps and reductions appear in the regions of strong velocity shears/vorticity between the head's Earthward and side tailward flows on head's two sides. There, ion agyrotropic motion occurred asymmetrically. Since vorticity on the duskside was opposite to the ion gyromotion, the penetrating from the dawnside higher‐energy ions are expected to experience braking and remain on the duskside. Opposite to that, the higher‐energy ions penetrating from the duskside into the dawnside might be accelerated by the dawnside vortex, which has the same sense of rotation as the ion gyromotion. Such ions might be able to gain sufficient velocity to escape from the dawnside vortex, for example, further dawnward. Thus, it is the penetration of the superthermal ions from the dawnside across the head to the duskside that is responsible for the enhancement in the observed ion temperature on the duskside of the head. In turn, the dawnside ion temperature decreases due to escape of higher energy ions from the dawnside vortex. This phenomenon is known as ***ω* ·** **b** asymmetry of ion FLR effects (Cerri, 2018; Del Sarto et al., 2016; Huba, 1996; Franci et al., 2016; Markovskii et al., 2006; Parashar & Matthaeus, 2016; Yang et al., 2017). The ***ω* ·** **b** asymmetry of BICI DFs is expected to complement the known ion FLR effects with non‐significant vorticity during DF interaction with Earth's dipolar field lines (Pritchett & Runov, 2017; Sergeev et al., 2009).

The ions' patterns were further modulated at smaller scales by the electromagnetic ion‐cyclotron waves. In the simulation, the waves were observed in the density trough/temperature hump regions in Figure 2. In THEMIS observations, the waves were identified as tailward ion populations around PHI_*i*_ = 180 (or alternatively modulated Earthward ion population near PHI_*i*_ = 0) around 12:12 UT in the second panel of Figure 1. The waves were responsible for local depressions of temperature in the temperature hump, as well as for local reduction of Earthward V_*X*_ and the negative E_*Z*_ hump (cf. Figures 1 and 4).

Both processes lead to the local current sheet deformations at their characteristic scales. At the head's duskside, the deformations started off‐equator locally compressing the current sheet. There, B_*Z*_ became significantly smaller and even negative (B_*Z*_ field was still positive everywhere at *z* = 0, indicating that there has been no onset of reconnection). Because such current sheet deformations progressed toward the neutral sheet plane with time (cf. Movie S1), the temperature hump formation and growth of electromagnetic ion‐cyclotron waves are potential mechanisms for forcing current sheet reconnection locally and in a competitive manner at a later stage of BICI development, as predicted by the earlier simulations (Pritchett & Coroniti, 2011, 2013).

## Supporting information

Supporting Information S1

Movie S1

## Data Availability

Data sets used for producing the figures are available at the website https://doi.org/10.6084/m9.figshare.16910098.v2.
