# Supplementary material for: Magnetotail Ion Structuring by Kinetic Ballooning‐Interchange Instability
Source: Geophys Res Lett. 2022 Feb 8;49(3):e2021GL096796. doi: 10.1029/2021GL096796 (PMC9286044; doi:10.1029/2021GL096796)
Supplement: Supplementary file 1 — Supporting Information S1 [file GRL-49-0-s001.pdf]

Supporting Information for "Magnetotail Ion Structuring by Kinetic Ballooning-Interchange Instability"

Evgeny V. Panov[1,2], San Lu[3], Philip L. Pritchett[4]

[1]Space Research Institute, Austrian Academy of Sciences, Graz, Austria
[2]Institute of Physics, University of Graz, Austria
[3]University of Science and Technology of China, Hefei, China
[4]Department of Physics and Astronomy, University of California, Los Angeles, CA, USA

Contents of this file

1. Figures S1-S6.
2. PIC Simulation Setup.

Additional Supporting Information (Files uploaded separately)

1. Caption for Movie S1.

Introduction

PIC Simulation Setup

The present investigation employs a 3-D PIC simulation model that retains the full dynamics for both electrons and ions. The initial magnetic field configuration is similar to those considered previously in [*Pritchett and Coroniti*, 2010, 2013] and is described by the vector potential $A_{0y}(x,z)$ given by

$$A_{0y}(x,z) = B_0 L \ln\{\cosh[F(x)(z/L)]/F(x)\}, \tag{1}$$

where $F(x)$ is a slowly varying but otherwise arbitrary function. For a nonconstant $F(x)$, there is a finite B_z field, which at the center of the current sheet has the form

$$B_{0z}(x,0) = -B_0 L F'(x)/F(x). \tag{2}$$

The specific configuration used in the present study is illustrated in Figure S6. Figure S6a shows the 2-D (x,z) magnetic field configuration, and Figure S6b shows the initial equatorial magnetic field profile $B_{0z}(x,0)$. The BICI modes will be excited in the region of the tailward gradient in B_z ($20 < x/\rho_{i0} < 28$), which corresponds to a region of decreasing entropy as x increases tailward [*Schindler and Birn*, 2004; *Pritchett and Coroniti*, 2013]. Here, ρ_{i0} is the ion gyroradius in the asymptotic lobe B_0 field.

Previous PIC simulations of BICI generation [*Pritchett and Coroniti*, 2010, 2013; *Pritchett et al.*, 2014] have considered a charge neutral, generalized Harris configuration in which the ion and electron cross-tail drifts are given by $V_{di} = -2cT_i/eB_0L$ and $V_{de} = 2cT_e/eB_0L$, where e is the magnitude of the fundamental electric charge and L is the half-width of the current sheet. In the present study, we consider a charged current sheet in which the electrons carry all of the cross-tail current with a net drift of $V_{de}^{ch} = 2c(T_i + T_e)/eB_0L$. Since the ions now carry no current, an electric field $E_{0z}(x,z)$ must be present in order to balance the nonuniform ion pressure,

$$E_{0z}(x,z) = -(2T_i/eL)F(x)\tanh[F(x)(z/L)]. \tag{3}$$

The (ion) density distribution is given by

$$n(x,z) = n_0 F^2(x)\text{sech}^2[F(x)(z/L)] + n_b, \tag{4}$$

where n_0 is the characteristic equatorial density at $x/\rho_{i0} = 16$ and $n_b = 0.08n_0$ is a constant background density. The density distribution for the current-carrying electrons is similar to that for the ions but with a larger characteristic density n_e given by $(n_e - n_0)/n_e = [T_i/(T_i + T_e)](V_{de}^{ch}/c)^2$.

The simulation has a grid $N_x \times N_y \times N_z = 512 \times 1024 \times 256$ distributed over the ranges $0 \le x/\rho_i \le 32, 0 \le y/\rho_{i0} \le 64, -8 \le z/\rho_{i0} \le 8$, so that $\rho_{i0} = 16\Delta$, where Δ is the grid spacing. The ion to electron mass ratio is $m_i/m_e = 64$, all particle temperatures are equal to $m_i V_A^2/4$ (here $V_A = (B_0^2/4\pi n_0 m_i)^{1/2}$ is a representative Alfvén speed), the electron plasma frequency/gyrofrequency ratio is $\sqrt{2}$, and the electron Debye length is Δ. The total number of particles in the simulation is 5.8 billion.

The simulation employs "closed" boundary conditions at the x boundaries [*Pritchett and Coroniti*, 1998]. No magnetic flux is allowed to cross these boundaries, corresponding to the condition $\delta E_y = 0$, and particles that cross these boundaries are reinserted back into the system in the opposite half z plane with $v_x = -v_x$ and $v_z = -v_z$ [*Pritchett et al.*, 1991]. In addition, the perturbed field δE_z is assumed to vanish at these boundaries. At the z boundaries, conducting conditions are assumed, and particles striking such a boundary are reintroduced in the opposite half z plane with $v_x = -v_x$ and $v_z = v_z$. This symmetric condition on the particle reflection is valid in the absence of a guide magnetic field. Periodicity in the y direction is assumed for both the particles and fields. The coordinate system used in the simulations has x increasing tailward (away from the Earth), y directed dawnward, and z directed northward. In the simulation figures, the coordinates are expressed in units of ρ_{i0}.

Caption for Movie S1.

Results from 3D PIC simulation of BICI development in the electron (charged) current sheet as seen in B_X (a), B_Y (b) and B_Z (c) magnetic field components, ion density (d), kinetic ion energy density (e), T_{XX} (f) and T_{XY} (g) ion temperature components, P_{XY} ion pressure tensor component (h), off-plane ion vorticity component ω_{iz} (i), and in-plane ion divergence $\partial V_{iX}/\partial x + \partial V_{iY}/\partial y$ (j) between $\Omega_{i0}t$=161 and $\Omega_{i0}t$=280. The contours of B_Z magnetic field component are overplotted as black curves. The star glyphs at (x/ρ_{i0},y/ρ_{i0},z/ρ_{i0})=(10.5,23.0,-1.5) (magenta), at (x/ρ_{i0},y/ρ_{i0},z/ρ_{i0})=(12.5,22.0,-1.5) (blue) and at (x/ρ_{i0},y/ρ_{i0},z/ρ_{i0})=(13.5,20.5,-1.5) (cyan) denote the location of three virtual spacecraft also discussed in Figure 3 of the manuscript.

References

Pritchett, P. L., and F. V. Coroniti (1998), Interchange instabilities and localized high-speed flows in the convectively-driven near-Earth plasma sheet, *in Substorms-4, edited by S. Kokubun and Y. Kamide, p. 443, Kluwer Acad. Publ., Dordrecht.*

Pritchett, P. L., and F. V. Coroniti (2010), A kinetic ballooning/interchange instability in the magnetotail, *J. Geophys. Res.*, *115*, A06301, doi:10.1029/2009JA014752.

Pritchett, P. L., and F. V. Coroniti (2013), Structure and consequences of the kinetic ballooning/interchange instability in the magnetotail, *J. Geophys. Res.*, *118*, 146–159, doi: 10.1029/2012JA018143.

Pritchett, P. L., F. V. Coroniti, R. Pellat, and H. Karimabadi (1991), Collisionless reconnection in two-dimensional magnetotail equilibria, *J. Geophys. Res.*, *96*, 11, doi: 10.1029/91JA01094.

Pritchett, P. L., F. V. Coroniti, and Y. Nishimura (2014), The kinetic ballooning/interchange instability as a source of dipolarization fronts and auroral streamers, *J. Geophys. Res.*, *119*, 4723–4739, doi:10.1002/2014JA019890.

Schindler, K., and J. Birn (2004), MHD stability of magnetotail equilibria including a background pressure, *J. Geophys. Res.*, *109*, A10208, doi:10.1029/2004JA010537.

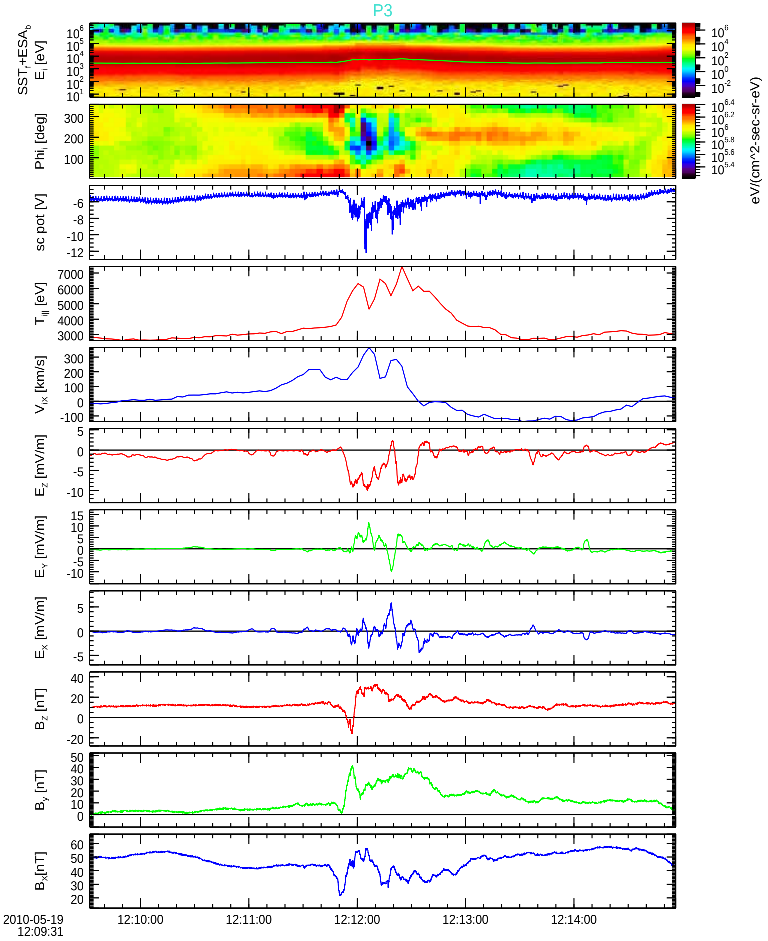

Figure S1. Time History of Events and Macroscale Interactions during Substorms probe P3 observations on 19 May 2010 between 12:09:30 and 12:15 UT. From top to bottom are shown: joint SST (full mode) and ESA (burst mode) ion differential flux spectra, azimuthal (PHI; 0 degrees corresponds to the Earthward direction) angle of ion motion, spacecraft potential (a high-resolution proxy to electron density), parallel component of the ion temperature (red), GSM V_X ion velocity component, DSL E_Z (red), E_Y (green) and E_X (blue) electric field components 3s-long-sliding-window averaged, GSM B_Z (red), B_Y (green) and B_X (blue) magnetic field components.

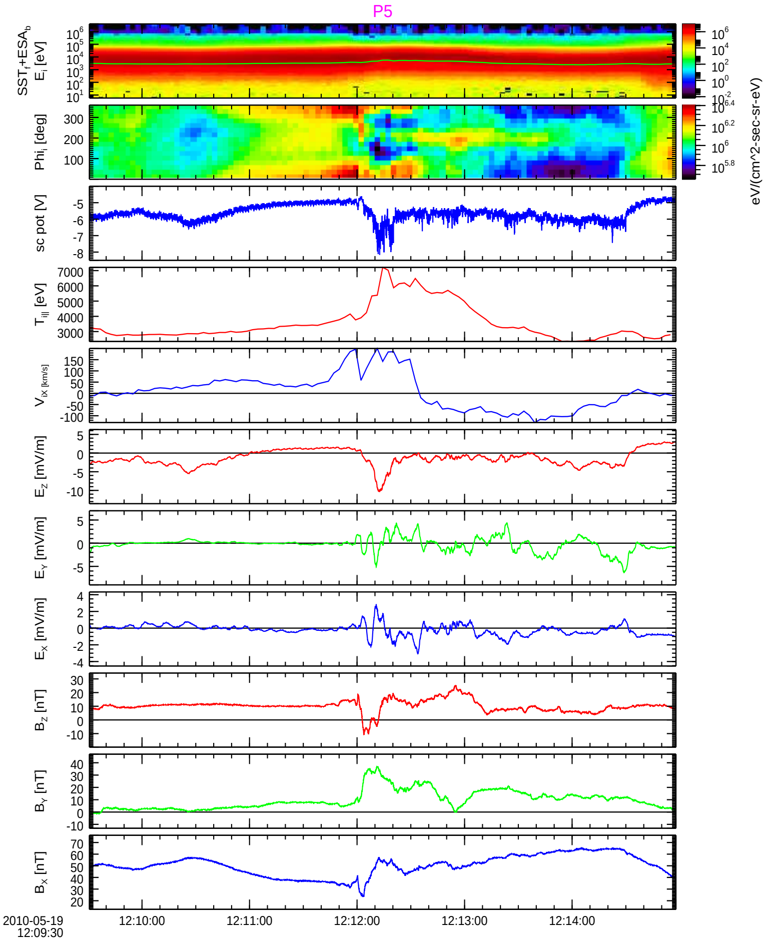

Figure S2. Time History of Events and Macroscale Interactions during Substorms probe P5 observations on 19 May 2010 between 12:09:30 and 12:15 UT. From top to bottom are shown: joint SST (full mode) and ESA (burst mode) ion differential flux spectra, azimuthal (PHI; 0 degrees corresponds to the Earthward direction) angle of ion motion, spacecraft potential (a high-resolution proxy to electron density), parallel component of the ion temperature (red), GSM V_X ion velocity component, DSL E_Z (red), E_Y (green) and E_X (blue) electric field components 3s-long-sliding-window averaged, GSM B_Z (red), B_Y (green) and B_X (blue) magnetic field components.

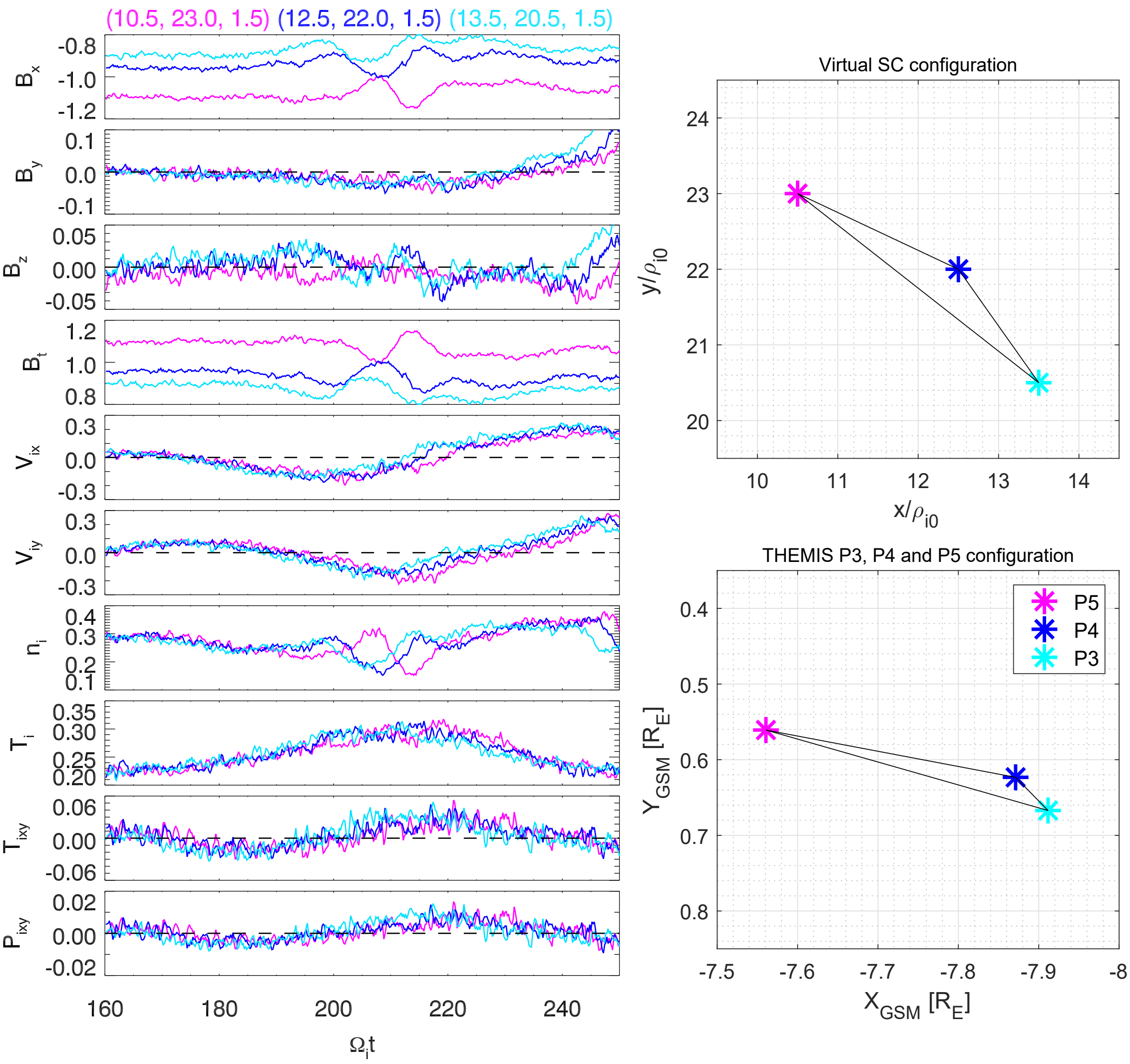

Figure S3. (left) Results from 3D PIC simulation of BICI development in the electron (charged) current sheet between $\Omega_{i0}t$=160 and $\Omega_{i0}t$=250 as seen in (from top to bottom) three magnetic field components and in the total magnetic field (B_x, B_y, B_z, B_t), X and Y ion velocity components V_{ix} and V_{iy}, ion density N_i, average ion temperature T_i, nondiagonal ion temperature and ion pressure tensor elements T_{ixy} and P_{ixy}. In the simulation, the X axis is directed antisuward and the Y axis is dawnward, which is opposite to the GSM X and Y axes. The three curves correspond to the location of three virtual spacecraft at (x/ρ_{i0},y/ρ_{i0},z/ρ_{i0})=(10.5,23.0,1.5) (magenta), at (x/ρ_{i0},y/ρ_{i0},z/ρ_{i0})=(12.5,22.0,1.5) (blue) and at (x/ρ_{i0},y/ρ_{i0},z/ρ_{i0})=(13.5,20.5,1.5) (cyan), i.e. at the same X and Y positions as in the left column of Figure 3 of the manuscript, but in the opposite Z-location. (right) xy configurations of three virtual spacecraft (top) and three THEMIS probes at 12:12UT (bottom).

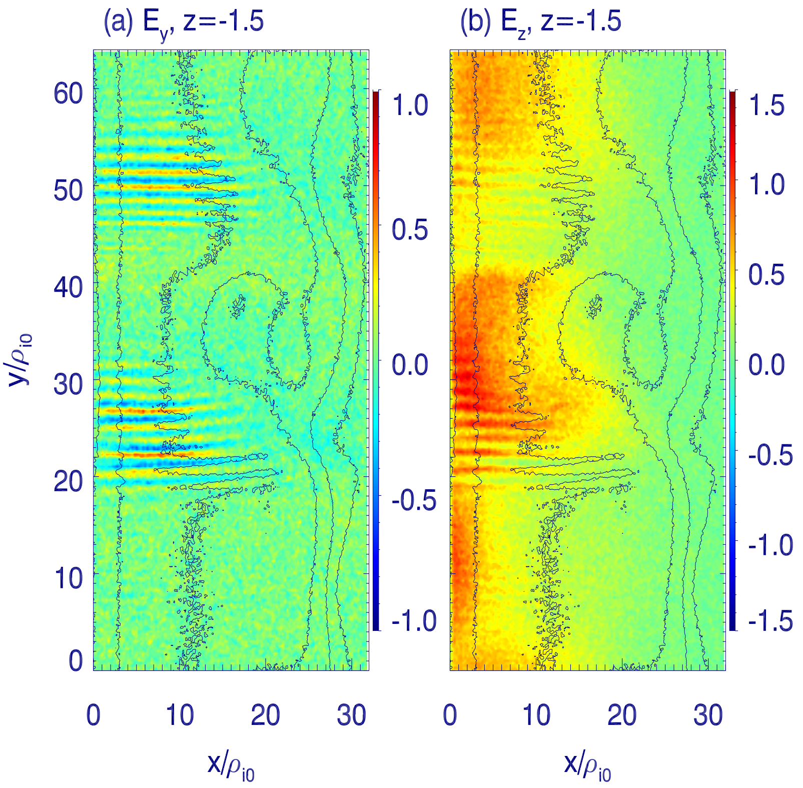

Figure S4. PIC simulated Ey (a), Ez (b) electric field components. The results are plotted in x, y plane cut at z/ρ_{i0} = -1.5 at time Ω_{i0t} = 210.

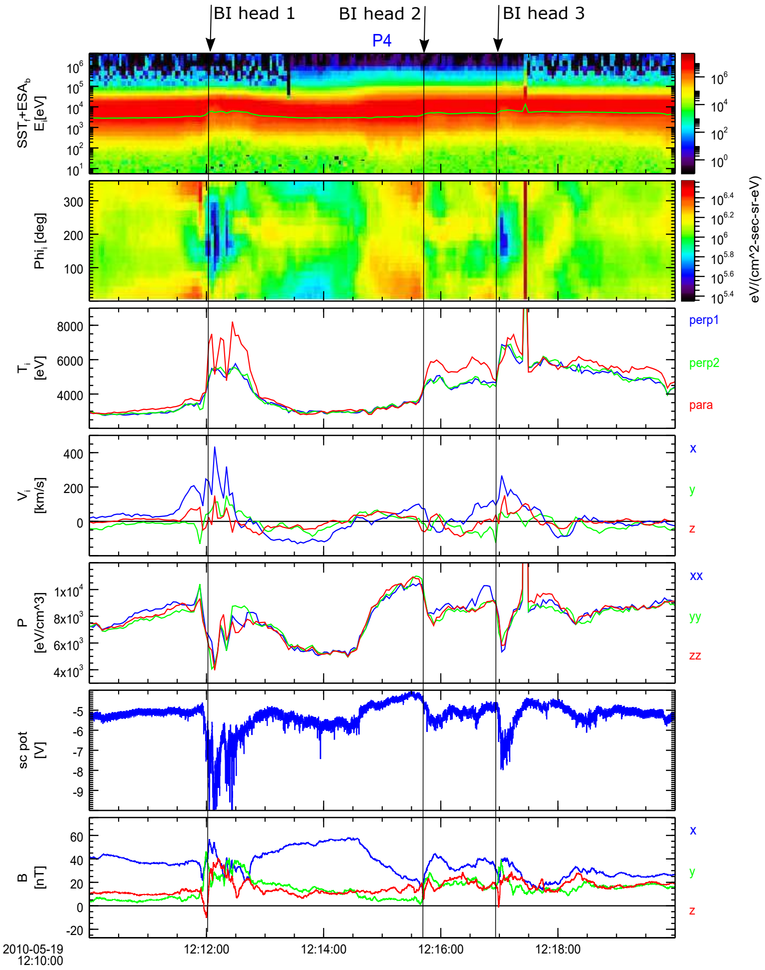

Figure S5. From top to bottom are shown Time History of Events and Macroscale Interactions during Substorms probe P4 observations on 19 May 2010 between 12:10:00 and 12:20 UT: joint SST (full mode) and ESA (burst mode) ion differential flux spectra, azimuthal (PHI; 0 degrees corresponds to the Earthward direction) angle of ion velocity, parallel (red) and perpendicular (blue and green) components of the ion temperature, three GSM components of the ion velocity V_i, three diagonal GSM components of the ion pressure tensor P_{xx} (blue), P_{yy} (green) and P_{zz} (red), spacecraft potential (a high-resolution proxy to electron density), and three GSM components of the magnetic field.

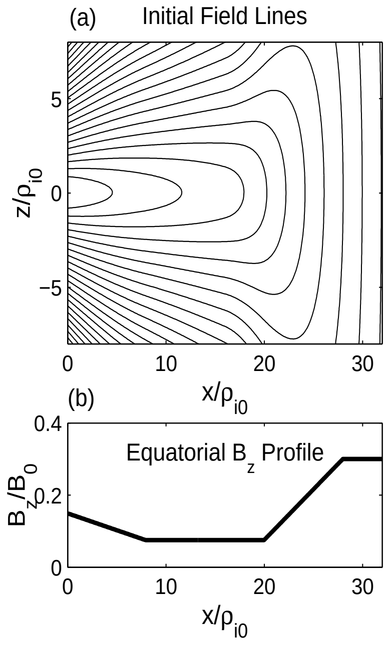

Figure S6. Specific initial PIC simulation configuration used in the present study: 2-D (x, z) magnetic field configuration (a), and initial equatorial magnetic field profile $B_{0z}(x, 0)$ (b).
